# Evaluation of pneumococcal and tetanus vaccine responses in patients with rheumatoid arthritis receiving baricitinib: results from a long-term extension trial substudy

**DOI:** 10.1186/s13075-019-1883-1

**Published:** 2019-04-18

**Authors:** Kevin L. Winthrop, Clifton O. Bingham, Wendy J. Komocsar, John Bradley, Maher Issa, Rena Klar, Cynthia E. Kartman

**Affiliations:** 1 Division of Infectious Diseases, Oregon Health Sciences University, Portland, OR USA; 2 Divisions of Rheumatology and Allergy and Clinical Immunology, Johns Hopkins University, Baltimore, MD USA; 3 Eli Lilly and Company, Indianapolis, IN USA; 4 IQVIA, Durham, NC USA

**Keywords:** Rheumatoid arthritis, Vaccination, DMARDs (biologics), Janus kinase inhibitors

## Abstract

**Background:**

Clinical guidelines recommend pneumococcal and tetanus vaccinations in patients with rheumatoid arthritis (RA). Baricitinib is an oral, selective Janus kinase (JAK) 1/JAK 2 inhibitor and is approved for the treatment of moderately to severely active RA in adults in over 50 countries including European countries, the USA, and Japan. This substudy evaluated pneumococcal conjugate and tetanus toxoid vaccine (TTV) responses in patients with RA receiving baricitinib. These vaccines elucidate predominantly T cell-dependent humoral antibody response.

**Methods:**

Eligible RA patients receiving baricitinib 2 mg or 4 mg with or without concomitant methotrexate (MTX) were enrolled in a phase 3 long-term extension trial (RA-BEYOND; ClinicalTrials.gov, NCT01885078) in USA/Puerto Rico. Patients were vaccinated with 13-serotype pneumococcal conjugate vaccine (PCV-13) and TTV. Primary endpoints were the proportion of patients achieving a satisfactory humoral response for PCV-13 (≥ 2-fold increase in anti-pneumococcal antibody concentrations in ≥ 6 serotypes) and TTV (≥ 4-fold increase in anti-tetanus concentrations) at 5 weeks post-vaccination. Secondary endpoints included humoral responses at 12 weeks and functional responses of serotypes 4, 6B, 14, and 23F (twofold and fourfold increases in opsonic indexes at 5 and 12 weeks).

**Results:**

Of 106 patients with a mean duration of RA of approximately 12 years, 80% were female, 30% were taking corticosteroids, and 89% (*N* = 94) were taking baricitinib plus MTX; most patients (97% PCV-13/96% TTV) completed the evaluations. Overall, 68% (95% CI 58.4, 76.2) of patients achieved a satisfactory response to PCV-13, 43% (34.0, 52.8) achieved a ≥ 4-fold increase in anti-tetanus concentrations, and 74% (64.2, 81.1) achieved a ≥ 2-fold increase. PCV-13 response was similar for patients taking corticosteroids (71%; 53.4, 83.9) vs those not (67%; 55.2, 76.5). The percentage of sera with a ≥ 2-fold increase in post-vaccination opsonic indexes at week 5 ranged from 47% (serotype 14) to 76% (serotype 6B). Through 12 weeks post-vaccination, seven patients (6.6%) reported injection-site events. There were no deaths during the substudy, and three patients experienced a serious adverse event.

**Conclusions:**

Approximately two thirds of patients on long-term baricitinib achieved satisfactory humoral and functional responses to PCV-13 vaccination, while TTV responses were less robust. PCV-13 response was not diminished in those taking concomitant corticosteroids.

**Trial registration:**

ClinicalTrials.gov, NCT01885078. Registered on 24 June 2013.

## Background

Patients with rheumatoid arthritis (RA) have an increased risk for a variety of infections due to diminished immune responses from their disease, associated comorbid conditions, and the immunosuppressive therapies used to treat RA [1–3]. Both RA management guidelines and recommendations from the Advisory Committee on Immunization Practices suggest vaccinating patients with RA against pneumococcal disease [4] with 13-serotype pneumococcal conjugate vaccine (PCV-13) and 23-serotype pneumococcal polysaccharide vaccine (PPSV-23) [5, 6]. There are no specific recommendations for tetanus toxoid vaccine (TTV) in patients with RA, but the CDC recommends that adults should receive a booster of tetanus and diphtheria every 10 years after the first dose of tetanus, diphtheria, and pertussis as a child or adult [5].

Baricitinib is an oral, selective inhibitor of Janus kinase (JAK)1 and JAK2 [7]. JAKs mediate signal transduction for a variety of cytokines [8], including those involved in T cell activation and proliferation, and inhibition of such pathways could theoretically diminish vaccine responses. Baricitinib is approved for the treatment of moderately to severely active RA in adults in over 50 countries including European countries, the USA, and Japan [9–12]. Given the mechanism of action of baricitinib, this study was conducted to evaluate the immunogenicity of the largely T cell-dependent vaccines PCV-13 and TTV in RA patients receiving treatment with baricitinib [13, 14].

## Methods

### Patients

Patients from the phase 3 long-term extension (LTE) trial for baricitinib (RA-BEYOND; NCT01885078) were invited to participate in this vaccine substudy. Patients in RA-BEYOND were 18 years or older with moderately to severely active RA and had completed the final active study treatment in one of the originating studies. In this substudy, patients were enrolled from the USA and Puerto Rico. Within the LTE, patients were receiving either 2-mg or 4-mg daily doses of baricitinib, and these doses were maintained during participation in the vaccine substudy. Inclusion criteria required that patients on methotrexate (MTX) or concomitant oral corticosteroids were on a stable dose for > 6 weeks prior to entry. Patients were excluded if they had a known allergy or hypersensitivity to any component of the vaccines, had received prior PPSV-23 within the last 12 months or prior PCV-13 at any time, had received prior TTV within the last 5 years, had recent (within 3 months) history of diagnosed pneumococcal infection or other infection requiring hospitalization, had a prior history of Guillain-Barre syndrome, or were using any of the following concomitant background conventional synthetic disease-modifying antirheumatic drugs (csDMARDs): cyclosporine, leflunomide, or azathioprine. Changes in allowed csDMARD therapy, use of intra-articular or oral corticosteroids, or baricitinib dose titration were not allowed unless required following rescue, which, in RA-BEYOND, was based on Clinical Disease Activity Index criteria at defined time points. The vaccine substudy was conducted in accordance with the ethical principles of the Declaration of Helsinki and Good Clinical Practice guidelines and was approved by each center’s institutional review board or ethics committee. All patients provided written informed consent.

### Study design

**Fig. 1:**
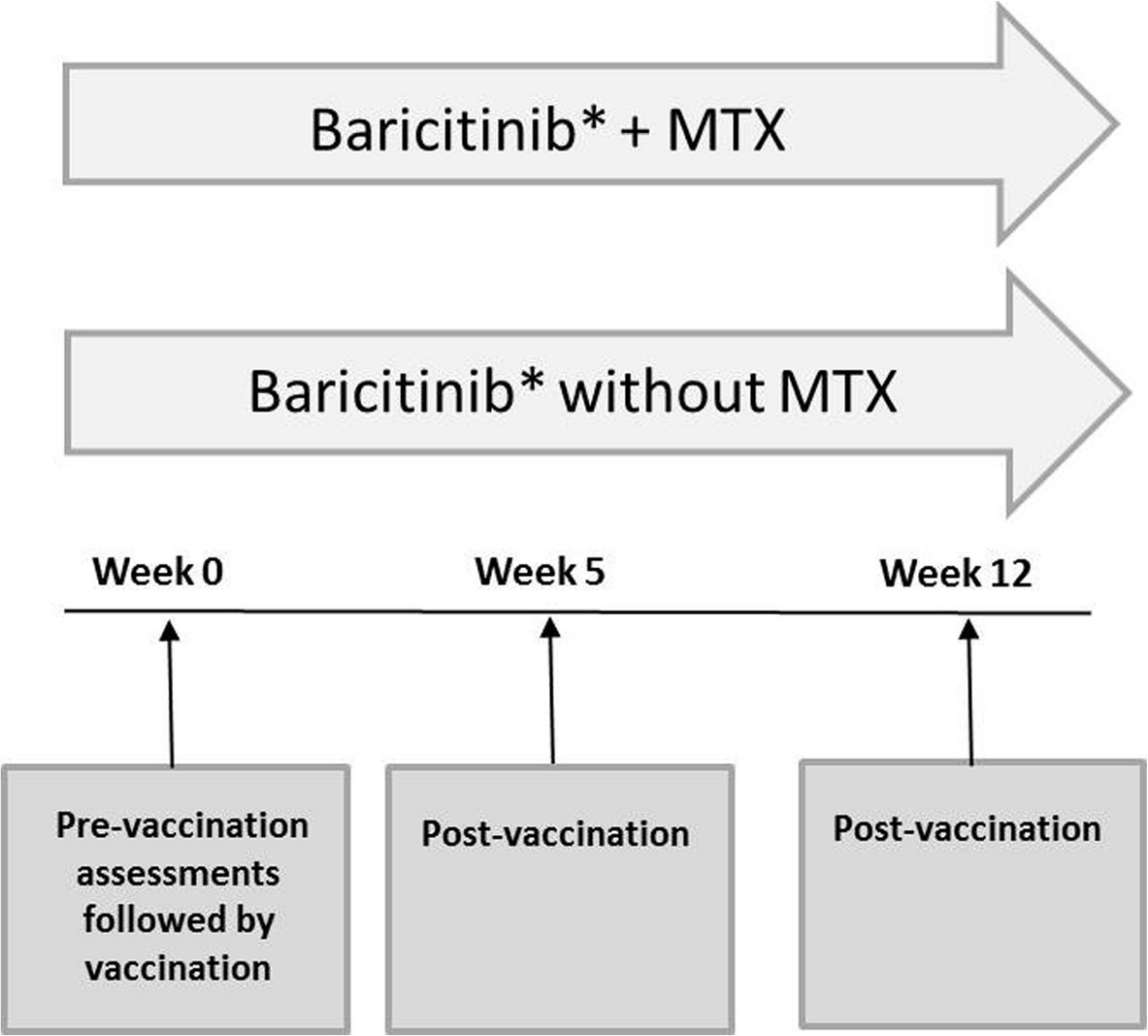
RA-BEYOND vaccine substudy design. Primary assessment was satisfactory humoral response to PCV-13 and TTV. *Background conventional synthetic disease-modifying antirheumatic drugs allowed except cyclosporine, azathioprine or leflunomide. MTX = methotrexate; PCV-13 = 13-serotype pneumococcal conjugate vaccine; TTV = tetanus toxoid vaccine

Enrolled patients were taking either baricitinib 2 mg or 4 mg in the LTE study (RA-BEYOND), and patients could continue on background MTX (Fig. 1). All patients received open-label, single-dose 0.5-mL intramuscular injections of PCV-13 (Prevnar 13®, Wyeth Pharmaceuticals, a subsidiary of Pfizer, Inc.) and TTV (Boostrix®, GlaxoSmithKline Biologicals) in opposite arms. The vaccines were used in accordance with their labels [15, 16] and immunization guidelines [5, 17]. Blood samples for measurement of anti-pneumococcal immunoglobulin G (IgG) and anti-tetanus IgG antibody concentrations were collected at the same time as regularly scheduled labs for the LTE; at the first visit of the vaccine substudy, the lab draw preceded vaccine administration (Fig. 1). Measurement of antibody concentrations were performed within the Q^2^ Solutions network at Focus Laboratories (San Juan Capistrano, CA, USA) using validated methods. In addition to the evaluation of anti-pneumococcal IgG concentrations, a functional assessment (opsonophagocytic activity) of IgG against four serotypes of *S. pneumoniae* (4, 6B, 14, and 23F) was also performed. The functional assay provides a more robust assessment of the protectiveness of an individual’s IgG titers. This measure has been used in prior pneumococcal vaccine studies [18, 19] and is likely particularly suited to understanding vaccine responses in the setting of immunocompromised patients where titers could be lower, yet still show functionality [20].

### Efficacy and safety measures

The primary outcome for each vaccine was the proportion of patients with satisfactory humoral responses at week 5. For the pneumococcal vaccine, satisfactory response was defined as a ≥ 2-fold increase from vaccination in antibody titers in ≥ 6 of the 13 pneumococcal serotypes (1, 3, 4, 5, 6A, 6B, 7F, 9V, 14, 18C, 19A, 19F, and 23F) [14, 21]. For tetanus vaccination, satisfactory response was defined as a ≥ 4-fold increase from vaccination in anti-tetanus concentration in patients with baseline anti-tetanus IgG concentration ≥ 0.1 IU/mL [21] These definitions of satisfactory response of two- or fourfold increase have been used in prior studies for both of these vaccines, as well as the polysaccharide pneumococcal vaccine [13, 22–27]. Secondary outcomes were geometric mean fold rise (GMFR) from baseline in pneumococcal serotype and tetanus antibodies at week 5. Exploratory measures included the primary and secondary outcomes at week 12 and TTV ≥ 2-fold increase in concentration at weeks 5 and 12. Safety assessments focused on injection-site events as well as routine safety monitoring within the LTE.

### Opsonophagocytic assays

Multiplexed opsonophagocytic activity assays were performed for pneumococcal serotypes 4, 6B, 14, and 23F (University of Alabama at Birmingham, Nahm Laboratory) as previously described [28]. Briefly, various dilutions of test sera (in duplicate) were incubated with bacteria (four strains) at room temperature for 30 min. Baby rabbit serum (final 12.5%) and HL60 cells (4 × 10^5^ cells/well) were added, and plates were incubated at 37 °C/5% CO_2_ for 45 min. Ten microliters of reaction mixture were spotted onto agar plates. An overlay agar containing 2,3,5-triphenyltetrazolium chloride and the selective antibiotic was added, and plates were incubated overnight at 37 °C/5% CO_2_. The number of surviving colonies was enumerated, and colony counts were converted to opsonization indexes (OIs), defined as the reciprocal of the interpolated dilution of serum that killed 50% of the target bacteria. Twofold and fourfold increases in OI at weeks 5 and 12 were evaluated [29]. Additionally, for each serotype tested, we assessed the association between ≥ 2-fold increases in anti-pneumococcal IgG responses and OI responses.

### Statistical analysis

Humoral response analyses were performed using the evaluable population for each vaccine, for each post-baseline time point. The evaluable population included patients who received a vaccination and pre- and post-vaccination antibody assays for a post-vaccination time point. For the co-primary objectives, the percentages of patients with a satisfactory humoral response at week 5 were summarized along with 95% confidence intervals (CIs), which were calculated based on the Wilson score method without continuity correction [30]. Because most patients (89%) were on concomitant MTX, we were unable to evaluate the potential influence of MTX on vaccine response. Results are presented for the overall baricitinib group (*N* = 106).

Analyses for secondary objectives were conducted using the evaluable vaccine populations at week 12 post-vaccination. GMFR post-vaccination and 95% CI were calculated at weeks 5 and 12 for each pneumococcal serotype IgG and anti-tetanus IgG, after taking the exponential of the mean difference in logarithm transformation concentration between pre- and post-vaccination using the paired *t* test. Subgroup analyses were conducted according to corticosteroid use at baseline (yes, no), age group at vaccination (< 65 years, ≥ 65 years), baricitinib dose (2 mg, 4 mg), number of csDMARDs including MTX taken at time of vaccination (0, 1, 2, ≥ 3), and Simplified Disease Activity Index (SDAI) at or prior to vaccination (≤ 3.3, > 3.3 and ≤ 11, > 11). A 95% CI was calculated if there were ≥ 5 patients in a subgroup and was based on the Wilson score method without continuity correction. The safety analysis set included all enrolled patients receiving at least one vaccination.

## Results

A total of 106 patients enrolled in the vaccine substudy. Patients had been in the parent LTE trial for up to 4 years at the time of vaccination. Most patients were female and white with a mean age of 55.1 years and mean duration of RA of 12.1 years; patients had good control of disease activity as measured by validated composite indexes including the SDAI (Table 1).

**Table 1:** Baseline demographics and disease characteristics

	Total baricitinib (*N* = 106)
Age, years, mean (SD)	55.1 (11.5)
< 65 years, *n* (%)	82 (77)
≥ 65 years, *n* (%)	24 (23)
Female, *n* (%)	85 (80)
Race, *n* (%)	
White	89 (84)
Black/African American	15 (14)
Asian	1 (1)
Multiple or other	1 (1)
Duration of RA diagnosis, years, mean (SD)	12.1 (9.3)
MTX use, *n* (%)	
Without MTX	12 (11)
MTX-treated	94 (89)
Weekly dose (mg/week), mean (SD)	18.2 (6.6)
Current corticosteroid use, *n* (%)	32 (30)
Daily dose (mg/day), mean (SD)	6.2 (2.7)
Weight, kg, mean (SD)	87.6 (21.7)
Swollen joint count, of 66, mean (SD)	3.7 (4.7)
Tender joint count, of 68, mean (SD)	5.0 (9.0)
hsCRP, mg/L, mean (SD)	9.3 (15.2)
HAQ-DI, mean (SD), (range 0–3)	0.9 (0.6)
DAS28-CRP, mean (SD) (range 2–10)	2.9 (1.1)
CDAI, mean (SD)	8.9 (8.8)
SDAI, mean (SD)	9.9 (8.8)
Physician’s global assessment of disease activity (0–100), mean (SD)	14.2 (14.6)

Demographic and disease characteristics are based on vaccine substudy baseline

*CDAI* Clinical Disease Activity Index, *DAS28* Disease Activity Score 28 Joints, *HAQ-DI* Health Assessment Questionnaire-Disability Index, *hsCRP* high-sensitivity C-reactive protein, *N* number of patients in the safety population, *MTX* methotrexate, *RA* rheumatoid arthritis, *SD* standard deviation, *SDAI* Simplified Disease Activity Index

Most patients (96.2%) completed both the 5- and 12-week visits. No patients discontinued participation due to adverse events. At week 5, 103 (97.2%) and 102 (96.2%) patients were evaluable for PCV-13 and TTV, respectively.

### Humoral responses at weeks 5 and 12

**Fig. 2:**
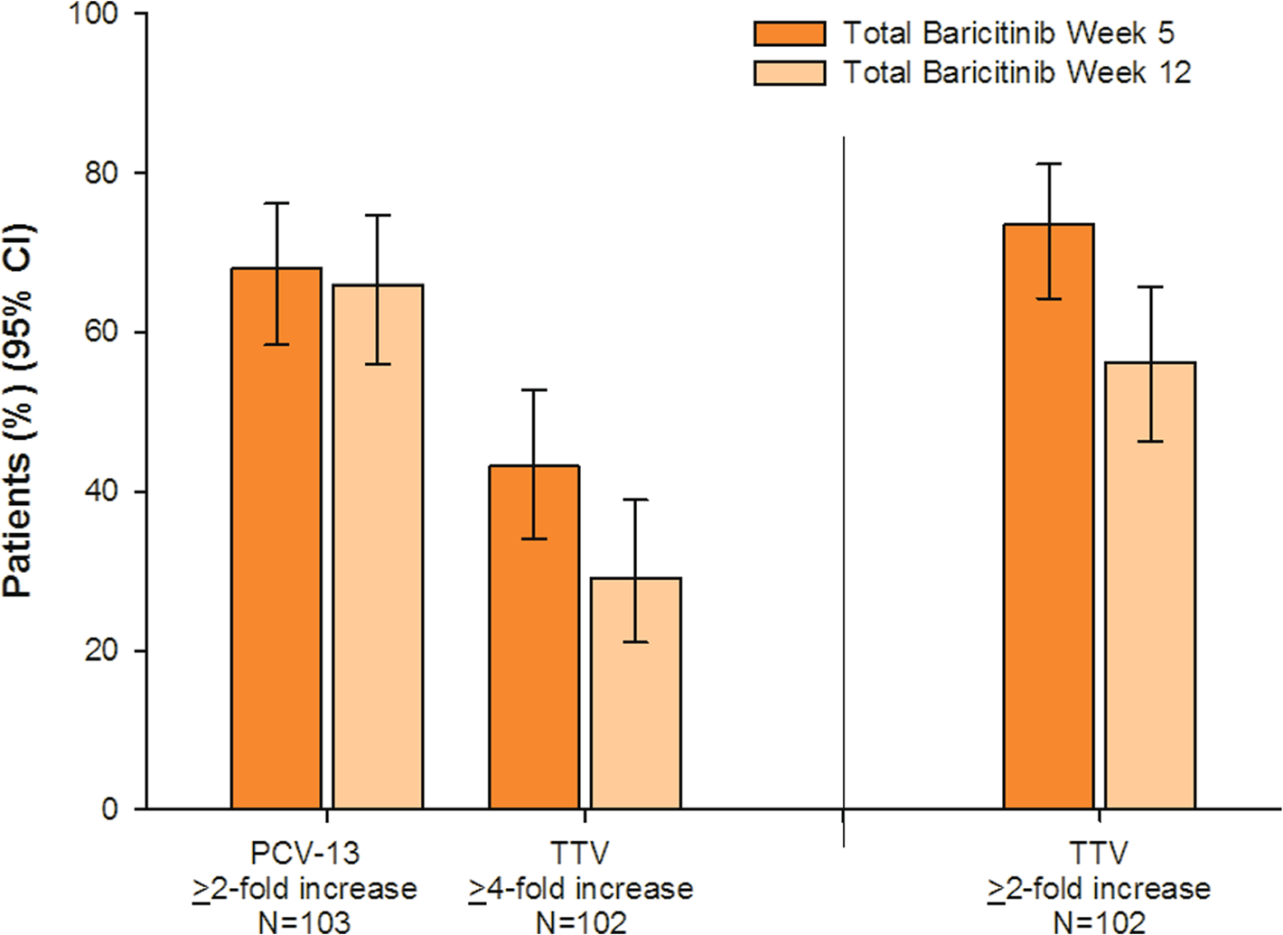
Satisfactory humoral responses at weeks 5 and 12. CI = confidence interval; IgG = immunoglobulin G; PCV-13 = 13-serotype pneumococcal conjugate vaccine; TTV = tetanus toxoid vaccine

For the PCV-13 vaccine at week 5, a majority of patients (68%) achieved a ≥ 2-fold increase in concentration in ≥ 6 serotypes; week 12 responses were similar to week 5 responses (Fig. 2). For TTV, all patients had a baseline anti-tetanus IgG concentration ≥ 0.1 IU/mL. Less than half of patients (43%) achieved ≥ 4-fold increase in concentration at week 5; a greater percentage of patients achieved a ≥ 2-fold concentration increase (74%). For TTV, both ≥ 2-fold and ≥ 4-fold week 12 responses were lower than week 5 responses).

Humoral response at week 5 varied across subgroups and between the pneumococcal and tetanus vaccines (Table 2). The percentage of patients with satisfactory responses was similar for PCV-13 regardless of a baricitinib 2-mg or 4-mg dose, concomitant corticosteroids, and SDAI response. However, for TTV, 33% (95% CI 15.2, 58.3) of patients taking baricitinib 2 mg showed a humoral response compared to 45% (95% CI 34.8, 55.3) of those taking baricitinib 4 mg; the percentages were 52% (95% CI 34.8, 68.0) and 39% (95% CI 28.9, 51.1) for those taking and not taking concomitant corticosteroids, respectively. For both pneumococcal and tetanus responses, patients ≥ 65 years old had lower response rates compared to younger patients. Analyses of humoral responses and GMFR to PCV-13 and TTV based on the number and type of csDMARDs (other than MTX) were limited by the small sample size.

**Table 2:** Number and proportions of patients who achieved humoral response at week 5 by subgroup

	PCV-13	TTV
Overall baricitinib group (*N* = 106)	70 (68)	44 (43)
Concomitant corticosteroids		
Yes (*N* = 31)	22 (71)	16 (52)
No (*N* = 72)	48 (67)	28 (39)
Age group		
Patients < 65 years (*N* = 80)	59 (74)	37 (46)
Patients ≥ 65 years (*N* = 23)	11 (48)	7 (32)
SDAI prior to vaccination		
≤ 3.3 (*N* = 21)	13 (62)	11 (55)
> 3.3 and ≤ 11 (*N* = 47)	34 (72)	20 (43)
> 11 (*N* = 32)	21 (66)	13 (41)
Baricitinib dose		
2 mg (*N* = 16)	11 (69)	5 (33)
4 mg (*N* = 87)	59 (68)	39 (45)

Data are reported as *n* (%). PCV-13 satisfactory humoral response defined as a ≥ 2-fold increase from baseline in antibody concentrations in ≥ 6 of the 13 pneumococcal serotypes. TTV satisfactory humoral response defined as a ≥ 4-fold increase from baseline in anti-tetanus concentration in patients with baseline anti-tetanus IgG concentration ≥ 0.1 IU/mL

*PCV-13* 13-serotype pneumococcal conjugate vaccine, *SDAI* Simplified Disease Activity Index, *TTV* tetanus toxoid vaccine

### IgG antibody concentrations and GMFR

**Fig. 3:**
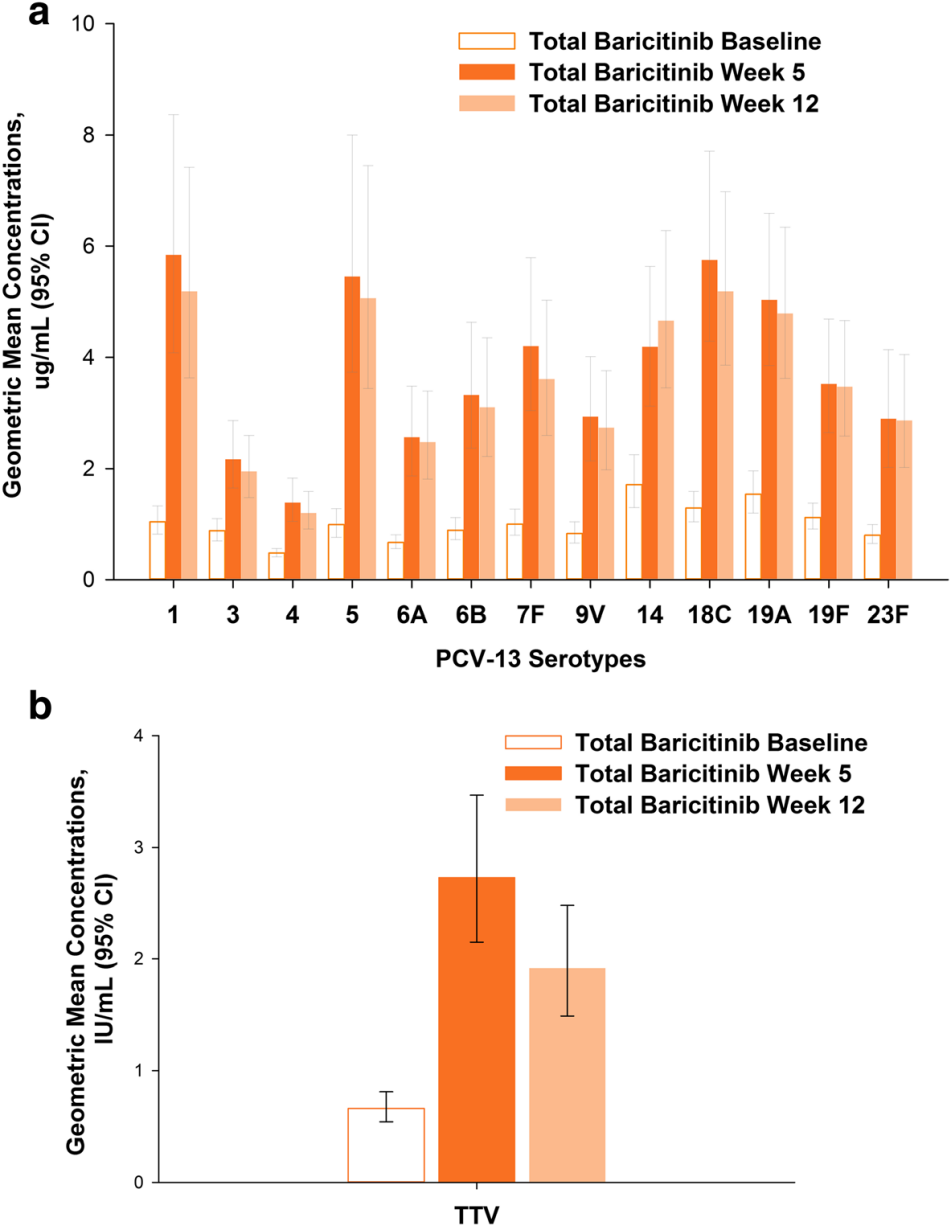
Concentrations for serotype-specific anti-pneumococcal (**a**) and anti-tetanus (**b**) IgG antibodies. All concentrations were significantly higher compared to baseline (*p* ≤ 0.001). CI = confidence interval; PCV-13 = 13-serotype pneumococcal conjugate vaccine; TTV = tetanus toxoid vaccine

**Fig. 4:**
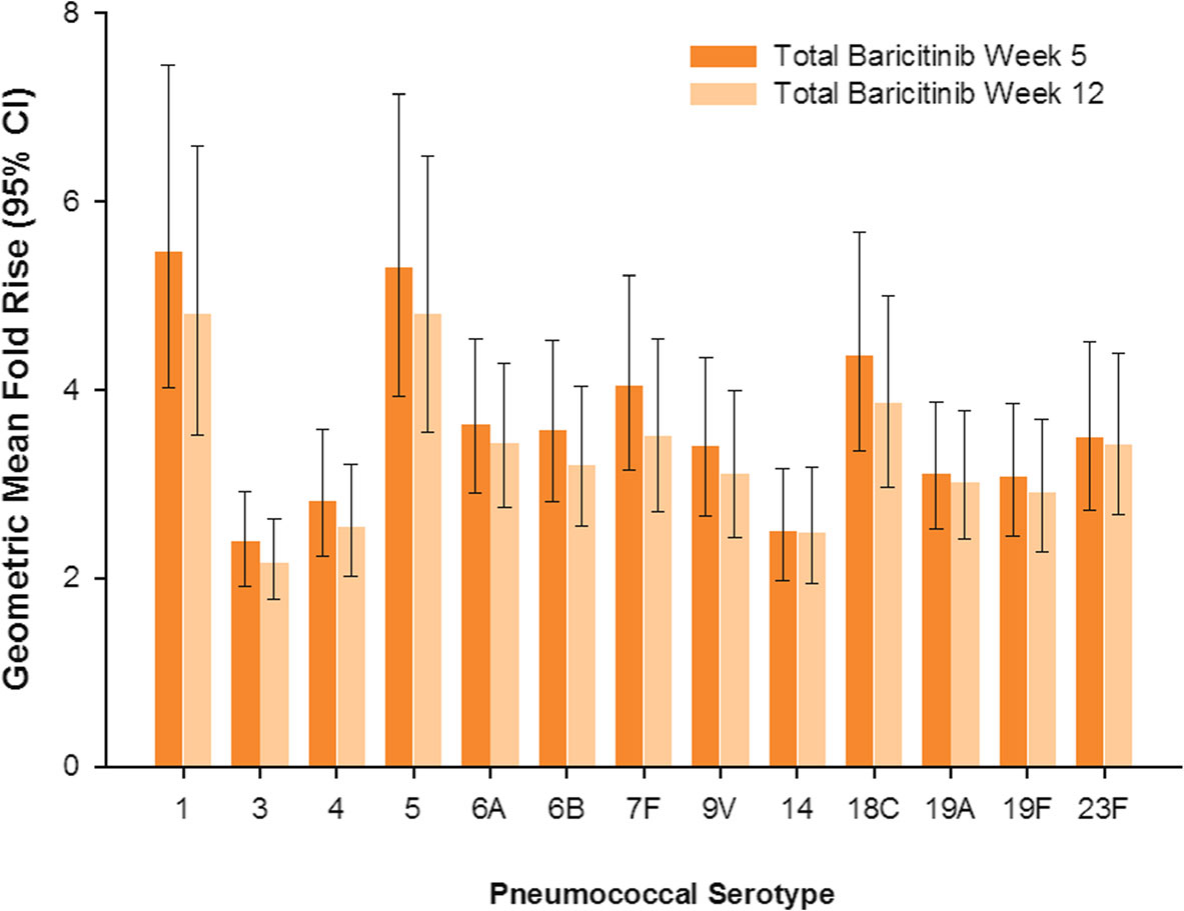
Pneumococcal serotype-specific geometric mean fold rise from baseline to week 5 and week 12. CI = confidence interval

In the total baricitinib-treated group the geometric mean concentrations for anti-pneumococcal IgG antibodies at 5 weeks post-vaccination varied from 1.39 μg/mL (serotype 4) to 5.84 μg/mL (serotype 1) compared to 0.48 μg/mL and 1.04 μg/mL at baseline, respectively (Fig. 3). Concentrations were significantly higher compared to baseline (*p* ≤ 0.001) for all serotypes at week 5 and week 12; the magnitude of antibody concentration elevation decreased slightly from week 5 to week 12. Similarly, the anti-tetanus IgG antibody concentrations were significantly higher at both post-baseline time points (*p* ≤ 0.001), with a decrease in the antibody concentration from week 5 to week 12 (Fig. 3). The GMFR responses ranged from approximately twofold to fivefold among the 13 serotypes (Fig. 4) and were approximately threefold to fourfold for TTV at both weeks 5 and 12.

### OI responses

**Fig. 5:**
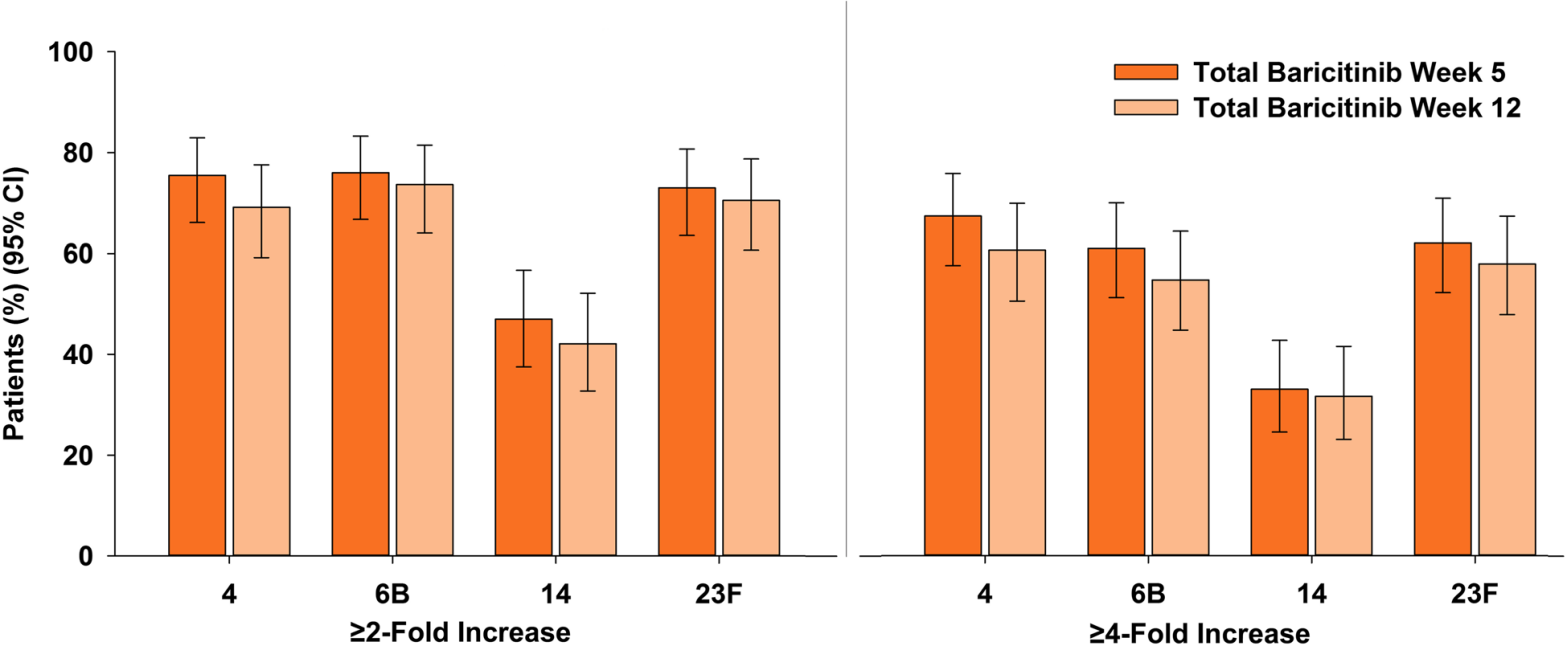
Percentage of patients with a ≥ 2-fold or ≥ 4-fold increase in opsonization index in four serotypes. CI = confidence interval

The percentage of patients with a ≥ 2-fold increase in post-vaccination OIs at week 5 ranged from 47.0% (serotype 14) to 76.0% (serotype 6B) (Fig. 5). Lower responses were found in older patients (≥ 65 years) for all four serotypes whereas other subgroups showed no consistent patterns between groups (Table 3). Geometric mean opsonophagocytic assay titers to the four evaluated serotypes were significantly increased at both weeks 5 and 12 post-vaccination compared to prior to vaccination, *p* ≤ 0.001 (Table 4).

**Table 3:** Number and proportions of patients with OI ≥ 2-fold increase at week 5 in PCV-13 serotypes by subgroup

	Serotype 4	Serotype 6B	Serotype 14	Serotype 23F
Overall baricitinib group (*N* = 106)	74 (76)	76 (76)	47 (47)	73 (73)
Concomitant corticosteroids				
Yes (*N* = 31)	27 (87)	26 (84)	14 (45)	20 (65)
No (*N* = 72)	47 (70)	50 (72)	33 (48)	53 (77)
Age group				
Patients < 65 years (*N* = 80)	61 (81)	66 (86)	40 (52)	58 (75)
Patients ≥ 65 years (N = 23)	13 (57)	10 (43)	7 (30)	15 (65)
SDAI prior to vaccination				
≤ 3.3 (*N* = 21)	14 (70)	16 (76)	11 (52)	16 (76)
> 3.3 and ≤ 11 (*N* = 47)	36 (82)	34 (76)	24 (53)	29 (64)
> 11 (*N* = 32)	23 (74)	25 (81)	11 (35)	26 (84)
Dose				
2 mg (*N* = 16)	11 (73)	11 (73)	9 (60)	9 (60)
4 mg (*N* = 87)	63 (76)	65 (76)	38 (45)	64 (75)

Data are reported as n (%)

OI opsonization index***,*** PCV-13 13-serotype pneumococcal conjugate vaccine***,*** SDAI Simple Disease Activity Index

**Table 4:** Geometric mean titers and fold increase in pneumococcal immunoglobulin and opsonophagocytic antibodies after PCV-13 vaccination

Baricitinib (*N* = 106)		Baseline	Week 5	Week 12
Immunoglobulin (μg/mL)				
Serotype 4	Geometric mean (95% CI)	0.48 (0.41, 0.56)	1.39 (1.05, 1.83)	1.20 (0.91, 1.59)
	Fold increase (95% CI)		2.8 (2.2, 3.6)*	2.6 (2.0, 3.2)*
Serotype 6B	Geometric mean (95% CI)	0.89 (0.72, 1.12)	3.32 (2.37, 4.63)	3.10 (2.22, 4.35)
	Fold increase (95% CI)		3.6 (2.8, 4.5)*	3.2 (2.6, 4.0)*
Serotype 14	Geometric mean (95% CI)	1.71 (1.30, 2.25)	4.19 (3.12, 5.64)	4.66 (3.45, 6.28)
	Fold increase (95% CI)		2.5 (2.0, 3.2)*	2.5 (1.9, 3.2)*
Serotype 23F	Geometric mean (95% CI)	0.80 (0.65, 0.99)	2.89 (2.02, 4.14)	2.86 (2.02, 4.05)
	Fold increase (95% CI)		3.5 (2.7, 4.5)*	3.4 (2.7, 4.4)*
OI				
Serotype 4	Geometric mean (95% CI)	92.1 (64.8131.0)	1717.9 (1166.0, 2531.1)	1016.8 (688.7, 1501.1)
	Fold increase (95% CI)		18.2 (11.8, 28.0)*	11.4 (7.5, 17.2)*
Serotype 6B	Geometric mean (95% CI)	153.8 (103.1, 229.4)	2153.0 (1431.3, 3238.7)	1519.0 (1029.2, 2241.9)
	Fold increase (95% CI)		13.2 (8.5, 20.4)*	9.0 (6.0, 13.4)*
Serotype 14	Geometric mean (95% CI)	270.2 (176.8, 413.1)	1328.2 (934.2, 1888.3)	1152.0 (811.6, 1635.2)
	Fold increase (95% CI)		4.9 (3.2, 7.5)*	3.8 (2.5, 5.7)*
Serotype 23F	Geometric mean (95% CI)	50.1 (35.1, 71.5)	627.2 (390.5, 1007.3)	489.3 (314.9, 760.3)
	Fold increase (95% CI)		12.1 (8.0, 18.5)*	9.3 (6.3, 13.8)*

*p ≤ 0.001 compared to baseline

*CI* confidence interval, *OI* opsonization index

### Association between serotype-specific pneumococcal IgG and OI responses

When examining all possible combinations of paired associations in terms of a ≥ 2-fold increase and < 2-fold increase between pneumococcal IgG and OI responses, a twofold increase in functional OI was observed in 15.0% (serotype 14) to 28.6% (serotype 4) of patients who did not have a twofold increase in pneumococcal IgG at week 5. The reverse was not observed, with only 3.0% (serotype 23F) to 8.0% (serotype 14) of patients without a twofold increase in functional OI having a ≥ 2-fold increase in IgG titer. As with other serotype-specific assessments, serotype 14 had lower response rates across all associations compared to the other serotypes (Table 5).

**Table 5:** Paired association between PCV-13 serotype-specific IgG and OI

	Serotype 4 *N* = 98	Serotype 6B *N* = 100	Serotype 14 *N* = 100	Serotype 23F *N* = 100
≥ 2-fold increase in pneumococcal IgG and functional OI	46 (47)	57 (57)	32 (32)	56 (56)
≥ 2-fold increase in pneumococcal IgG, but not functional OI	4 (4)	6 (6)	8 (8)	3 (3)
≥ 2-fold increase in functional OI, but not pneumococcal IgG	28 (29)	19 (19)	15 (15)	17 (17)
< 2-fold increase in both pneumococcal IgG and functional OI	20 (20)	18 (18)	45 (45)	24 (24)

Data are reported as *n* (%)

*IgG* immunoglobulin G, *OI* opsonization index, *PCV-13* 13-serotype pneumococcal conjugate vaccine

### Safety

Overall, there were 30 (28.3%) adverse events during the vaccine substudy. Seven patients (6.6%) reported injection-site events, which included pain and erythema; two patients reported moderate pain (one patient with PCV-13 and TTV vaccine and one patient with TTV vaccine). No deaths were reported during the vaccine substudy. Three patients (2.8%) experienced a serious adverse event during their participation in the vaccine substudy, but these events were not considered related to vaccine administration.

## Discussion

In this substudy of an ongoing phase 3 LTE in patients with RA, we evaluated the effect of baricitinib on vaccine responses to PCV-13 and TTV. At 5 weeks post-vaccination, we observed approximately two thirds of patients to have satisfactory humoral responses to PCV-13, while responses to TTV were somewhat less robust. Lower vaccine responses in RA populations are generally expected, depending on RA disease control, as compared to the healthy adults [31]. Responses to PCV-13 were maintained through week 12, while we observed some diminishment in humoral responses to TTV over this time period. Importantly, we found little or no negative effects of corticosteroid use on PCV-13 titer rise or functional antibody responses. Further, we identified a subset of individuals that lacked satisfactory IgG response, but in whom a satisfactory functional response was observed. This suggests that some patients identified as having poor PCV-13 vaccine responses still generate functional responses that are likely protective. There are few studies evaluating immune responses to PCV-13 within the inflammatory disease setting. Conversely, a number of studies have been performed evaluating responses to PPSV-23 in the context of biologic therapy. Our findings are similar to those reported for pneumococcal vaccine in the context of biologics, as well as TTV [13, 22, 23, 32]. First, for PCV-13, there is only one other study evaluating responses in those using JAK inhibitors, although it is a study of patients with psoriasis. The majority of patients in this study mounted an adequate response to PCV-13, with > 80% patients achieving measurable titers for each of the 13 serotypes and 60% mounting a > 4-fold response to TTV [24]. While a number of factors would likely explain the greater immunogenicity seen in this study (e.g., patients were younger, had a different underlying disease, and there was very little or no concomitant csDMARD or corticosteroid use), both this study and ours observed reasonably high proportions of patients mounting satisfactory responses to conjugate vaccines while receiving JAK inhibitors. For PPSV-23, a number of studies have been conducted among RA patients. With anti-TNF therapy, for example, the majority of patients mount satisfactory responses and such therapy seems to interfere little with the immunogenicity of the vaccine. In one study with RA patients using etanercept, there was an overall 2.6-fold increase in IgG antibody levels 4 weeks after vaccination. Other studies have examined RA patients using tofacitinib, where PPSV-23 responses ranged from 45 to 89% [22] and tocilizumab+MTX where PPSV-23 responses were 60% [23]. Published literature has shown that MTX can further decrease antibody response following vaccination with 7-serotype pneumococcal conjugate vaccine (PCV-7) [25, 26] or PCV-13 [27] when administered with background rituximab, abatacept, or tocilizumab; concomitant MTX when administered with tofacitinib can decrease antibody response to pneumococcal vaccine (PPSV-23) and to a lesser extent, influenza vaccine [22]. In addition, among RA patients taking rituximab and tocilizumab, TTV responses were 39% and 42% respectively, similar to that observed in our study [13, 23].

Functional responses to pneumococcal vaccine have received little prior study among RA patients. One such study evaluated RA patients taking abatacept and found a positive OI response for serotype 6B in some patients who did not have optimum IgG responses [18]. These findings were similar to our study in which we assessed four serotypes and also found that some baricitinib-treated patients mounted OI responses when an IgG response was not present. These results demonstrate that satisfactory functional pneumococcal antibody response could be attained even in some patients who lack an adequate IgG titer response and suggest the importance of measuring functional antibody responses in vaccine studies, particularly in the setting of immunosuppression where antibody titer responses could be less robust.

There were several limitations to this study. Because it was an LTE subpopulation and the sample size for the baricitinib 2-mg dose was too small for analysis, there was no control group for evaluation. Also, in our study, we were limited in evaluating the effect of MTX on vaccine response, due to the small size of the non-MTX group. Small sample size also hampered some of the other planned subgroup analyses. Additionally, current guidelines recommend administration of PPSV-23 following vaccination with PCV-13 for optimal vaccine response [5, 17] which was not within the scope of this substudy.

## Conclusions

In this sample of patients from the USA and Puerto Rico, administration of pneumococcal and tetanus vaccines during long-term treatment with baricitinib was well tolerated. Satisfactory pneumococcal humoral responses were demonstrated with both baricitinib 2 mg and 4 mg and were not affected by concomitant low-dose corticosteroid usage while TTV responses were less robust. Additionally, sustained functional response of the pneumococcal antibodies was demonstrated at week 5 and week 12.

## Abbreviations

CI: Confidence interval
csDMARD: Conventional synthetic disease-modifying antirheumatic drug
GMFR: Geometric mean fold rise
IgG: Immunoglobulin G
JAK: Janus kinase
LTE: Long-term extension
MTX: Methotrexate
OI: Opsonization index
PCV-13: 13-serotype pneumococcal conjugate vaccine
PCV-7: 7-serotpye pneumococcal conjugate vaccine
PPSV-23: 23-serotype pneumococcal polysaccharide vaccine
RA: Rheumatoid arthritis
SDAI: Simplified Disease Activity Index
TTV: Tetanus toxoid vaccine
